# ITN Mixtures of Chlorfenapyr (Pyrrole) and Alphacypermethrin (Pyrethroid) for Control of Pyrethroid Resistant *Anopheles arabiensis* and *Culex quinquefasciatus*

**DOI:** 10.1371/journal.pone.0055781

**Published:** 2013-02-07

**Authors:** Richard M. Oxborough, Jovin Kitau, Johnson Matowo, Emmanuel Feston, Rajab Mndeme, Franklin W. Mosha, Mark W. Rowland

**Affiliations:** 1 Department of Disease Control, London School of Hygiene and Tropical Medicine (LSHTM), London, United Kingdom; 2 Department of Entomology and Parasitology, Kilimanjaro Christian Medical University College (KCMUCo) of Tumaini University, Moshi, Kilimanjaro, Tanzania; 3 Department of Entomology, Pan-African Malaria Vector Research Consortium, (PAMVERC), Moshi, Kilimanjaro, Tanzania; National Institute for Communicable Diseases/NHLS, South Africa

## Abstract

Pyrethroid resistant *Anopheles gambiae* malaria vectors are widespread throughout sub-Saharan Africa and continued efficacy of pyrethroid ITNs is under threat. Chlorfenapyr is a promising pyrrole insecticide with a unique mechanism of action conferring no cross-resistance to existing public health insecticides. Mixtures of chlorfenapyr (CFP) and alphacypermethrin (alpha) may provide additional benefits over chlorfenapyr or alphacypermethrin used alone. An ITN mixture of CFP 100 mg/m^2^+alpha 25 mg/m^2^ was compared with CFP 100 mg/m^2^ and alpha 25 mg/m^2^ in a small-scale experimental hut trial in an area of wild *An. arabiensis*. The same treatments were evaluated in tunnel tests against insectary-reared pyrethroid susceptible and resistant *Culex quinquefasciatus*. Performance was measured in terms of insecticide-induced mortality, and blood-feeding inhibition. Tunnel tests showed that mixtures of CFP 100+ alpha 25 were 1.2 and 1.5 times more effective at killing susceptible *Cx. quinquefasciatus* than either Alpha 25 (P = 0.001) or CFP 100 (P = 0.001) ITNs. Mixtures of CFP100+ alpha 25 were 2.2 and 1.2 times more effective against resistant *Cx. quinquefasciatus* than either alpha 25 (P = 0.001) or CFP100 (P = 0.003) ITNs. CFP 100+ alpha 25 produced higher levels of blood-feeding inhibition than CFP alone for susceptible (94 vs 46%, P = 0.001) and resistant (84 vs 53%, P = 0.001) strains. In experimental huts the mixture of CFP 100+ Alpha 25 killed 58% of *An. arabiensis*, compared with 50% for alpha and 49% for CFP, though the differences were not significant. Blood-feeding inhibition was highest in the mixture with a 76% reduction compared to the untreated net (P = 0.001). ITN mixtures of chlorfenapyr and alphacypermethrin should restore effective control of resistant populations of *An. gambiae* malaria vectors, provide protection from blood-feeding, and may have benefits for resistance management, particularly in areas with low or moderate frequency of pyrethroid resistance. A wash-resistant mixture should be developed urgently.

## Introduction

From 2008–2010, 294 million insecticide-treated mosquito nets (ITNs) were supplied for use in sub-Saharan Africa, mainly through mass distribution campaigns. The rapid scale up of ITN distribution has resulted in an estimated 50% of households owning at least one ITN in 2011 compared with only 3% in 2000 [1]. ITNs are highly effective in reducing child mortality and incidence of uncomplicated and severe malaria [2]. Recent examples of significant decline in malaria incidence following ITN distribution include Kenya [3], Eritrea [4], Zanzibar [5], Burkina Faso [6], Rwanda and Ethiopia [7].

Pyrethroids are the only insecticides that are currently recommended for ITNs [8]. Pyrethroids have been the chemical of choice for malaria vector control in recent decades because of relatively low toxicity to humans, rapid knock-down of mosquitoes, prevention of blood-feeding through excito-repellency, long duration, and low cost. Use of pyrethroids in agriculture has been linked with the development and spread of pyrethroid resistance in *Anopheles gambiae* malaria vectors [9]–[11]. Rapid scaling up of pyrethroid ITNs and IRS for malaria vector control in Africa has probably accelerated the spread of resistance [12], [13]. As a consequence, 27 sub-Saharan African countries reported pyrethroid resistance in *An. gambiae* in 2011 [1].

Target site insensitivity and metabolic resistance mechanisms are widespread in *An. gambiae s.l.* across Africa and the effectiveness of long-lasting insecticide treated mosquito nets (LLINs) and indoor residual spraying of houses (IRS) with pyrethroids is under threat [14]. Experimental hut trials in Benin, an area of high frequency pyrethroid resistance, showed that holed pyrethroid ITNs failed to protect sleepers from being bitten and no longer had a mass killing effect on malaria vectors [15]. In community use, the level of insecticide resistance at which malaria control is compromised remains uncertain. In spite of resistance, vector control interventions may retain a degree of effectiveness, particularly as mosquitoes become less resistant with increasing age [16], [17].

Nevertheless, if LLINs and IRS are to remain effective it is essential that new public health insecticides are developed to address the growing problem of resistance [18]. The small market size and uncertainty of the public health insecticide market has limited commercial investment [19]. Despite added impetus for the development of new public health insecticides, notably from Innovative Vector Control Consortium (IVCC), alternative classes of insecticide for public health use are emerging slowly [19].

Several studies in Benin, Tanzania and India have demonstrated that chlorfenapyr (CFP) IRS and ITN are effective at controlling pyrethroid resistant malaria vectors [20]–[24]. CFP ITN at a dosage of 100 mg/m^2^ provided greater control of *An. gambiae s.s.* than pyrethroids in Benin (54% vs. 30% lambdacyhalothrin 18 mg/m^2^) but provided little protection, with blood-feeding inhibition of <5% [15], [22]. In this trial an ITN mixture of CFP and alphacypermethrin (alpha) was evaluated against pyrethroid resistant *Cx. quinquefasciatus* and wild *An. arabiensis*.

## Methods

### Mosquito Strains

*Cx. quinquefasciatus* Muheza is an insectary reared strain that is resistant to pyrethroids but susceptible to organophosphates (OPs) and carbamates. The strain is originally from Muheza, coastal Tanzania and has been reared since the 1990s. At Kilimanjaro Christian Medical University College (KCMUCo) this strain was selected for several generations with technical grade permethrin at the 3^rd^/4^th^ larval instar and is now strongly pyrethroid resistant (Table 1).

**Table 1:** Resistance status of *Cx. quinquefasciatus* Muheza strain.

Insecticide	Dosage	Number tested	% Mortality	95% Confidence Interval
Lambdacyhalothrin	0.05%	105	40	(31–49)
Permethrin	0.75%	310	21	(16–26)
Bendiocarb	0.10%	200	96	(93–99)
Malathion	5%	200	100	(100–100)

Percentage mortality of *Cx. quinquefasciatus* Muheza strain after exposure in World Health Organization (WHO) resistance tests lined with treated papers at diagnostic concentrations.

To characterize resistance mechanisms *Cx. quinquefasciatus* Muheza were exposed for 60 minutes in bottle bioassays, based on CDC protocols [25]. Probit analysis (Poloplus 1.0, LeOra software) was done to determine KDT-50 values (time for 50% of mosquitoes to be knocked down) and resistance ratios (Table 2). For the Muheza strain KDT-50 values were 57 and 56 mins when permethrin was mixed with PBO or DEF respectively, compared with 78 mins for permthrin alone. This indicated metabolic based resistance with involvement of non-specific esterases (NSEs) and mixed function oxidases (MFOs). Despite addition of PBO and DEF to permethrin there was a resistance ratio of 2.1 compared to the susceptible TPRI strain (Table 2). There was probably an additional mechanism present such as knockdown resistance (kdr).

**Table 2:** Summary of Bottle Bioassay Results; KDT-50 and Resistance Ratios for *Cx. quinquefasciatus* TRI (susceptible) and Muheza strains (resistant).

Insecticide	Dosage	Number tested	% Mortality	95% Confidence Intervals
Lambdacyhalothrin	0.05%	208	97	(95–99)
Permethrin	0.75%	207	100	(100–100)
Bendiocarb	0.10%	200	99	(98–100)
Malathion	5%	200	100	(100–100)

Time taken (minutes) for 50% of mosquitoes to be knocked-down (KDT-50) during exposure in 250 ml glass bottles treated with 10 µg/ml permethrin alone (no synergist) or mixed with synergists PBO, DEF or PBO+DEF (n = 100 for each treatment).

*Culex quinquefasciatus* TPRI is an insectary reared pyrethroid, OP, and carbamate susceptible strain that was taken from Tropical Pesticide Research Institute (TPRI) in Arusha to KCMUCo in 2006 (Table 3). *Anopheles arabiensis* were tested as 1^st^ generation offspring (F1) of wild collected adult mosquitoes from cattle sheds in Lower Moshi. Resistance testing using WHO cylinders in November 2011 shortly prior to the experimental hut trial showed resistance to pyrethoids but full susceptibility to dichlorodiphenyltrichloroethane (DDT) and fenitrothion (Table 4). After the experimental hut trial a sub-sample of 80 *An. arabiensis,* that were collected alive from the hut with alpha ITN, were tested for the presence of L1014F and L10145S kdr variants. No kdr mutations were detected, confirming earlier reports of metabolic resistance mechanisms [26].

**Table 3:** Resistance status of *Cx. quinquefasciatus* TPRI strain.

Mosquito strain	Permethrin (µg/ml)	Synergist (µg/ml)	KDT-50 (95% CI)	Resistance Ratio (95% CI)
TPRI	10	None	25 (24–26)	–
Muheza	10	None	78 (69–93)	3.1 (2.7–3.6)
Muheza	10	PBO 400	57 (53–61)	2.3 (2.1–2.5)
Muheza	10	DEF 125	56 (53–61)	2.3 (2.1–2.5)
Muheza	10	PBO 400+ DEF 125	51 (48–55)	2.1 (1.9–2.2)

Percentage mortality of *Cx. quinquefasciatus* TPRI strain after exposure in cylinder bioassays lined with treated papers at diagnostic concentrations.

**Table 4:** Resistance status of *Anopheles arabiensis* F1 strain.

Insecticide	Dosage	Number tested	% Mortality	95% Confidence Interval
Lambdacyhalothrin	0.05%	508	58	(54–62)
Permethrin	0.75%	490	76	(72–80)
DDT	4.00%	280	100	(100–100)
Fenitrothion	1.00%	195	100	(100–100)

Percentage mortality of *An. arabiensis* F1 wild strain after exposure in cylinder bioassays lined with treated papers at diagnostic concentrations.

### Insecticide Formulations and Dosages

The following insecticide formulations were used for all tests: Chlorfenapyr 21.45%, Lot Number 0134S03CD, BASF (Phantom suspension concentrate (SC), BASF Agricultural Products, Limburgerhof, Germany); Alphacypermethrin 6%, BASF (Fendona SC; BASF Agricultural Products, Limburgerhof, Germany).

***Tunnel Tests***

- 1- Untreated
- 2- Chlorfenapyr 100 mg/m^2^
- 3- Chlorfenapyr 200 mg/m^2^
- 4- Alphacypermethrin 25 mg/m^2^
- 5- Chlorfenapyr 100 mg/m^2^+Alphacypermethrin 25 mg/m^2^
- 6- Chlorfenapyr 200 mg/m^2^+Alphacypermethrin 25 mg/m^2^

***Experimental Hut Trial***

- 1- Untreated
- 2- Chlorfenapyr 100 mg/m^2^
- 3- Alphacypermethrin 25 mg/m^2^
- 4- Chlorfenapyr 100 mg/m^2^+Alphacypermethrin 25 mg/m^2^

40 denier polyester netting was hand dipped in insecticide solution based on WHO protocols [27]. Chlorfenapyr at 100 mg/m^2^ dosage was selected for hut trials as previous trials in Tanzania showed no significant increase in efficacy for dosages higher than 100 mg/m^2^ [21]. The alphacypermethrin dosage (25 mg/m^2^) was selected based on WHOPES recommended dosage range 20–40 mg/m^2^ [8].

### Tunnel Test

The tunnel test is designed to allow expression of the behavioural interactions that occur between free-flying mosquitoes and ITN during host-seeking. Cone bioassays are not suitable for this purpose because they are based on a fixed exposure time that may not be representative of exposure under natural conditions. Tunnel tests allow expression of host seeking behaviour at night which results in more realistic contact time with netting. Tunnel tests were carried out as a forerunner to hut trials to provide information on repellency, blood-feeding inhibition, and mortality. The equipment consisted of a square glass cylinder (25 cm in height, 25 cm in width, and 60 cm in length) divided into two sections by means of a netting-covered frame fitted into a slot across the tunnel [28]. In one of the sections, a guinea pig was housed unconstrained in a small wooden cage, and in the other section 50 unfed female mosquitoes aged 5–8 days were released at dusk and left overnight. Mosquitoes of the same age were divided between the treatments for each replicate. The netting surface was 400 cm^2^ and deliberately had nine 1-cm holes to give opportunity for mosquitoes to pass into the baited chamber. Netting treatments were tested simultaneously alongside an untreated negative control. The next morning, the numbers of mosquitoes found alive or dead, fed or unfed, in each section were scored. Live mosquitoes were removed from the sections, and held in paper cups under controlled conditions (25–27°C and 75–85% RH) and given access to sugar solution, and monitored for delayed mortality up to 72 h. *Cx. quinquefasciatus* mosquitoes were chosen for tunnel tests as a model insect due to availability of insectary-reared pyrethroid resistant and susceptible strains.

### Experimental Hut Trial

An experimental hut trial was conducted at KCMUCo Field Station in Lower Moshi Rice Irrigation Zone (3°22′S, 37°19′E) where *An. arabiensis* was the major malaria vector [24]. *An. arabiensis* densities were heavily dependent on rice cropping cycles with flooded rice fields being the breeding site. Experimental huts were constructed to a design described by the World Health Organization [27] and based on the original verandah-hut design developed in Tanzania in the 1960s [29], [30]. Minor modifications were made involving a) reduction of eave gap to 5 cm, b) addition of inner ceiling board, c) concrete floor surrounded by a water filled moat [21]. Wooden eave baffles were installed to prevent egress of mosquitoes that had entered the hut. The working principle of these huts has been described previously [21], [31].

There were 4 experimental huts in total; in 3 huts there was a different insecticide treated net and 1 hut had an untreated net. An adult volunteer slept in each hut nightly from 20∶30–6∶30. The 4 volunteers were rotated between huts on successive nights to reduce any bias due to differences in individual attractiveness to mosquitoes. Mosquito net treatments were rotated between huts after every 5^th^ night (4 trial nights and 1 non-trial night for cleaning and aeration with no net) for a total duration of 20 nights (16 trial nights). Mosquito collections were done using mouth-aspirators between 6∶30–08∶00 each morning by trained field assistants. White sheets were laid on the concrete floor to make dead mosquitoes more easily visible. Dead mosquitoes were collected from the floor of 2 closed verandahs, bedroom and 2 window traps. Live mosquitoes were collected from 2 closed verandahs, bedroom, and 2 window traps. Live mosquitoes were transferred to 150 ml paper cups and provided with 10% glucose solution for scoring delayed mortality after 24, 48, 72 h. Gonotrophic status was recorded as unfed, blood-fed, semi-gravid, or gravid. All members of the *An. gambiae* species complex identified by morphological characteristics were assumed to be *An. arabiensis* based on recent PCR identification [32]–[34].

### Ball Bioassay

All ITNs used in the experimental hut trial were tested 2 days before the trial started to assess toxicity against wild F1 *An. arabiensis*. Testing methodology was based on WHO protocols for testing ITNs [27]. Ball bioassays were done with 3 and 30 minutes exposure. 3 minutes is the standard WHO specified exposure time for pyrethroid nets, where as a prolonged exposure of 30 minutes may be more suitable for a non-repellent insecticide such as chlorfenapyr.

### Analysis

#### Tunnel tests

Data was entered into an Excel database and transferred to Stata 11 for data processing and analysis (Stata Corp LP, College Station, TX, USA). The outcomes of interest were proportion of mosquitoes penetrating the treated net, blood-feeding, and dead (i.e. total number of mosquitoes dead immediately plus delayed mortality after holding for a total of 72 h). Logistic regression for grouped data was used to estimate the outcomes, within each mosquito species, comparing results for treated and untreated nets clustering by replicate.

#### Experimental hut trials

The principal aim was to compare the efficacy of different types of ITN (pyrethroid, chlorfenapyr and mixture) as compared to a negative-control untreated net. The outcomes of interest were proportion of mosquitoes blood-feeding, dying (i.e. total number of mosquitoes dead immediately plus delayed mortality after holding for a total of 72 h) and exiting on successive nights. Logistic regression for grouped data was used to estimate the outcomes, within each trial, comparing results for treated and untreated nets clustering by day and adjusting for variation between individual sleepers and huts. Negative binomial regression was used to analyse numbers entering the huts (% deterrence).

### Ethics Statement

Institutional approval was obtained from: London School of Hygiene and Tropical Medicine, London, UK; Kilimanjaro Christian Medical University College of Tumaini University (KCMUCo), Moshi, Tanzania. The study was approved by the national ethics committee of Tanzania NIMR/HQ/R.8c/Vol.I/24. Written consent was obtained from all volunteer sleepers.

## Results

### Tunnel Tests

#### Pyrethroid Susceptible *Culex quinquefasciatus*

CFP 100 and CFP 200 mg/m^2^ produced similar levels of blood-feeding (37 and 42% respectively) and a percentage mortality (52, 60%) after 72 h that was slightly but significantly higher (P = 0.034) for CFP 200 (Table 5). Alpha produced significantly greater mortality 77% than either CFP 100 (P = 0.001) or CFP 200 (P = 0.001), and blood-feeding was lower at only 2% (P = 0.001 and 0.001 respectively). Mixtures of either CFP 100 or CFP 200 mg/m^2^ with alpha resulted in mortality significantly greater than alpha alone (93 vs 77%, P = 0.001; 99 vs 77%, P = 0.001) and blood-feeding levels significantly lower than CFP alone (42 vs 5%, P = 0.001; 37 vs 1%, P = 0.001).

**Table 5:** Tunnel test results for pyrethroid susceptible *Cx. quinquefasciatus* TPRI strain.

Insecticide mg/m^2^	N	% Mortality				% Penetration	% Blood-feeding	% Blood-fed of Penetrated	% Blood-fed & alive
		0h	24h	48h	72h				
Untreated	340	0^a^	2^a^	5^a^	7^a^	78^a^	77^a^	99^a^	71^a^
CFP 100	363	14^b^	44^b^	46^b^	52^b^	48^b^	42^b^	88^b^	41^b^
CFP 200	297	16 ^b^	42^b^	56^c^	60^c^	46^b^	37^b^	80^c^	29^b^
Alpha 25	351	61^c^	74^c^	74^d^	77^d^	26^c^	2^c^	8^d^	0^c^
CFP 100+ Alpha 25	350	74^d^	89^d^	91^e^	93^e^	14^d^	5^d^	36^e^	1^c^
CFP 200+ Alpha 25	340	87^e^	99^e^	99^f^	99^f^	11^d^	1^c^	9^d^	0^c^

Comparison of results for ITNs treated with CFP alone (100–200), alpha alone (25), and mixtures of CFP (100/200)+Alpha (25). If the superscript in a column is the same there was no significant difference between treatments (P>0.05).

#### Pyrethroid resistant *Culex quinquefasciatus*

**Figure 1:**
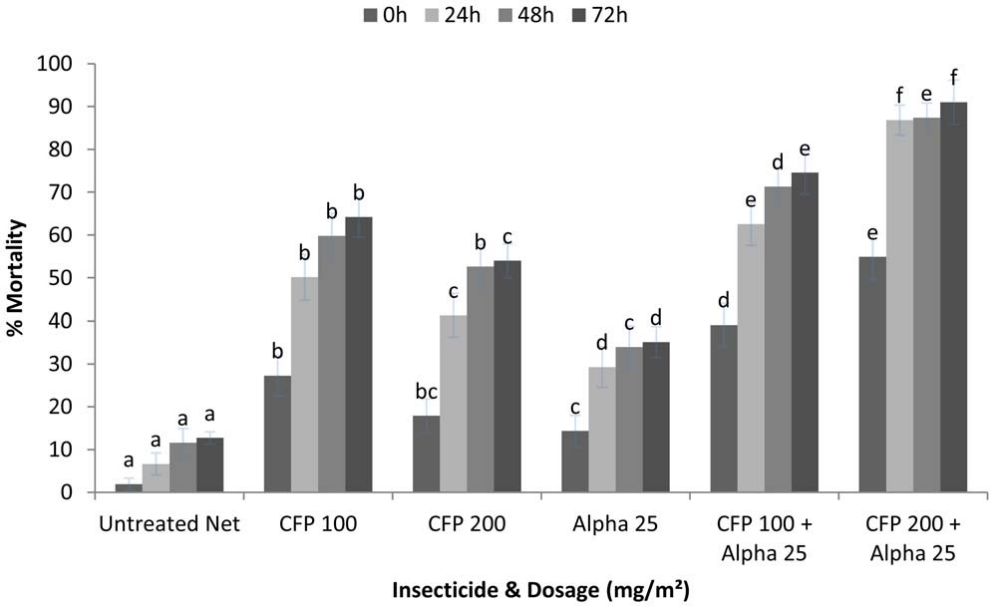
Percentage mortality of pyrethroid resistant *Cx. quinquefasciatus* in tunnel tests. Percentage mortality for ITNs treated with CFP alone (100 and 200), alpha alone (25), and mixtures of CFP (100/200)+alpha (25). If the superscript in a time period (0 h, 24 h, 48 h, 72 h) is the same there was no significant difference between treatments (P>0.05) (n = 350 p/treatment).

CFP 100 killed a significantly greater proportion than CFP 200 with 64 and 54% mortality respectively (P = 0.006) (Figure 1). Both dosages of CFP killed a greater proportion than alpha which only killed 35% (CFP 100 P = 0.001; CFP 200 P = 0.001). Mixtures of either CFP 100 or CFP 200 with alpha were more effective at killing *Cx. quinquefasciatus* than CFP alone (P_100_ = 0.003, P_200_ = 0.001) or alpha alone (P_100_ = 0.001, P_200_ = 0.001). The mixture of CFP 200+ alpha 25 was more effective than CFP 100+ alpha 25 with 91% mortality compared with 75% (P = 0.001).

**Figure 2:**
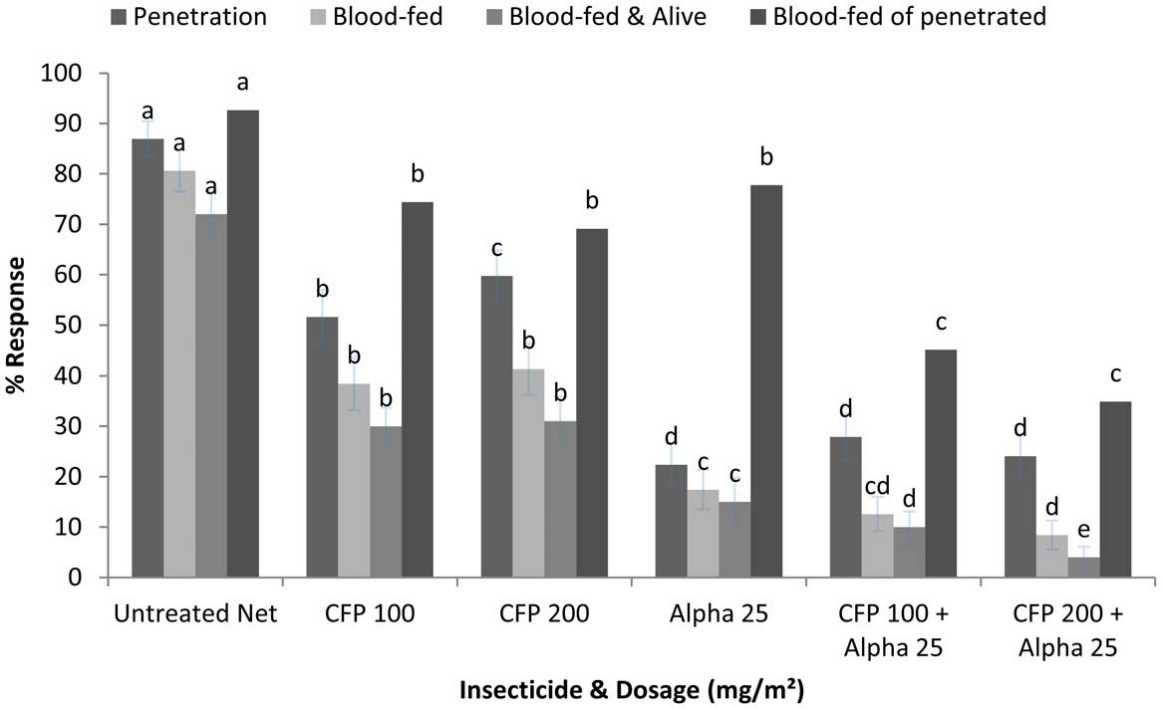
Percentage response of pyrethroid susceptible *Cx. quinquefasciatus* in tunnel tests. Percentage response for parameters related to repellency and blood-feeding for ITNs treated with CFP alone (100–200), alpha alone (25), and mixtures of CFP (100/200)+Alpha (25). If the superscript in a time period (0 h, 24 h, 48 h, 72 h) is the same there was no significant difference between treatments (P>0.05) (n = 350 p/treatment).

The majority of mosquitoes penetrated (87%) the untreated net and subsequently blood-fed (81%) and survived for 72 h (72%) (Figure 2). CFP 100 and 200 had a moderate effect with reduced penetration (52%, 60%, P = 0.029) and blood-feeding (38%, 41%, P = 0.431), with no significant difference between dosages. Alpha was more effective at reducing penetration (22%), and blood-feeding (17%) than CFP (P<0.05). Mixtures of either CFP 100 or CFP 200 with alpha resulted in similar levels of penetration (24%, 28%) and blood-feeding (8%, 13%) as alpha. Mixtures produced a significant reduction in the proportion blood-fed and alive at 72 h with 10% and 4% for CFP100+ alpha and CFP200+ alpha compared with 15% for alpha alone (P = 0.045 and 0.001, respectively).

### Experimental Huts

#### *An. arabiensis*

**Figure 3:**
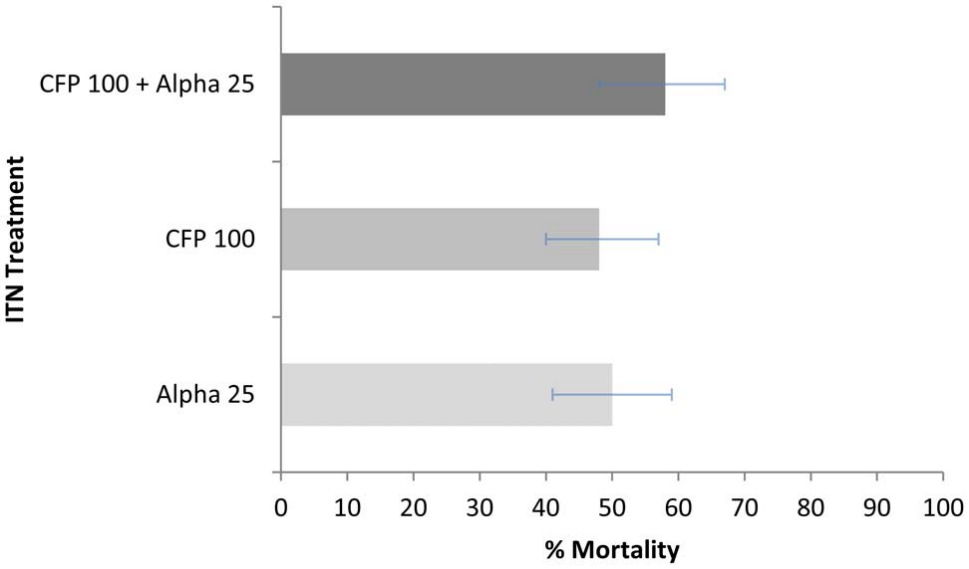
Percentage Mortality of *An. arabiensis* in Experimental Huts. Percentage mortality of *An. arabiensis* collected in experimental huts with ITNs treated with alpha 25, CFP 100, and a mixture of CFP 100+ Alpha 25.

The ITN mixture killed 58% of *An. arabiensis* after 72 h, but this was not significantly greater than CFP (P = 0.22) or alpha (P = 0.23). Levels of mortality were similar for CFP ITN (48%) and alpha ITN (50%), (P = 0.97), (Figure 3).

**Figure 4:**
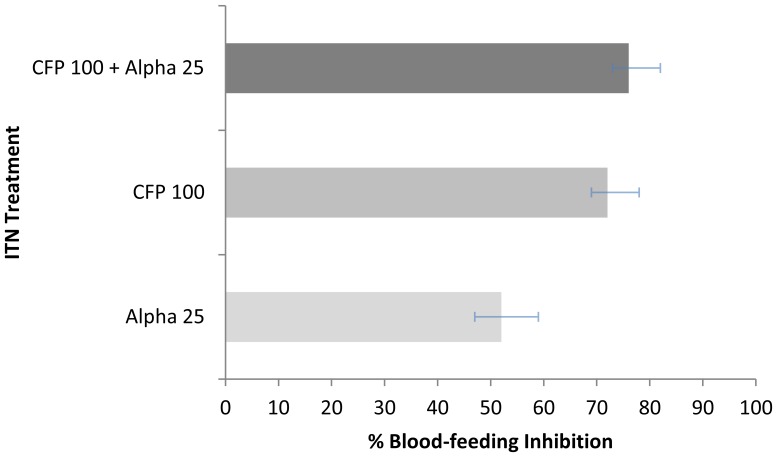
Percentage Blood-feeding inhibition of *An. arabiensis* in Experimental Huts. Percentage blood-feeding inhibition of *An. arabiensis* collected in experimental huts with ITNs treated with alpha 25, CFP 100, and a mixture of CFP 100+ Alpha 25.

The proportion of *An. arabiensis* that blood-fed on a volunteer sleeper with an untreated net (25%) was significantly higher than all treated nets (Alpha = 12%, P = 0.007; CFP = 7%, P = 0.001; Mixture = 6%, P = 0.001). The CFP 100+ alpha 25 mixture produced similar levels of blood-feeding inhibition (76%) to CFP (72%, P = 0.59) and neither differed to alpha (52%, P = 0.12), (Figure 4).

By the time of early morning mosquito collections 87% of *An. arabiensis* had exited the bedroom of the untreated net and collected either in the veranda or window trap. The mixture net (87%) produced significantly lower exiting rates compared to the untreated net (76%, P = 0.049). Most mortality for all ITN treatments had occurred by the morning of mosquito collections with very little delayed mortality between 24–72 h (Table 6).

**Table 6:** Experimental hut results for *An. arabiensis*.

Insecticide Dosage (mg/m^2^)	N	Mortality %				Exophily	Blood-feeding %	Blood-fed & Alive at 72h %
		Morning	24h	48h	72h			
Untreated	143^a^	14^b^	15^b^	15^b^	15^b^	87^b^	25^b^	24^b^
		(9–21)	(10–22)	(10–22)	(10–22)	(81–92)	(19–33)	(18–32)
CFP 100	135^a^	47^a^	47^a^	48^a^	48^a^	84^ab^	7^a^	6^a^
		(39–56)	(39–56)	(40–57)	(40–57)	(76–89)	(4–13)	(3–11)
Alpha 25	110^a^	45^a^	50^a^	50^a^	50^a^	84^ab^	12^a^	9^a^
		(36–54)	(41–59)	(41–59)	(41–59)	(76–89)	(7–19)	(5–16)
CFP 100+ Alpha 25	106^a^	52^a^	55^a^	58^a^	58^a^	76^a^	6^a^	5^a^
		(42–61)	(45–64)	(48–67)	(48–67)	(67–84)	(3–12)	(2–11)

Comparison of *An. arabiensis* results for ITNs treated with CFP 100, alpha 25, and mixture of CFP 100+ alpha 25. If the superscript in a column is the same there was no significant difference between treatments (P>0.05).

### Ball Bioassay Results

**Figure 5:**
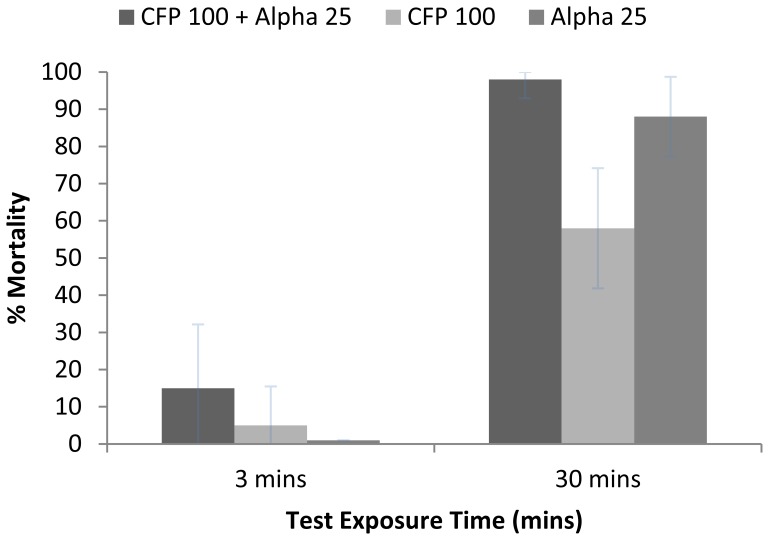
Ball Bioassay results for *An. arabiensis* using 3 and 30 minutes exposure. Results of ball bioassay (% mortality after 72 h holding) for mixture of CFP 100+ Alpha 25, CFP 100, and alpha 25 with *An. arabiensis F1 wild* and exposure time of 3 and 30 minutes.

3 minutes exposure time resulted in mortality rates of <15% for all treated nets (Figure 5). Prolonged exposure of 30 minutes resulted in 58% mortality for CFP 100, compared with 88% for alpha and 98% for the mixture. More than 90% of mortality occurred within 24 h after exposure for all treated nets.

## Discussion

The rationale for combining CFP and alpha as an ITN mixture was to;

- Restore effective control of pyrethroid resistant *An. gambiae* and *Cx. quinquefasciatus*.
- Achieve mortality rates that are higher than the single actives (CFP or alpha).
- Provide greater levels of personal protection than CFP ITN.
- Delay development of insecticide resistance in mosquito populations that are susceptible to both alpha and CFP.

In tunnel tests mortality was low for alpha against resistant *Cx. quinquefasciastus,* but mixtures of CFP 100/200+ alpha 25 were highly effective. The CFP component should restore control of all resistant mosquito populations due to its novel mode of action of disrupting respiration pathways (oxidative phosphorylation) in mitochondria [35], and lack of cross-resistance to known mechanisms in malaria vectors [24], [36], [37].

Mortality rates for mixtures of CFP 100/200+ alpha 25 against *Cx. quinquefasciatus* were greater than for CFP or alpha alone in tunnel tests. In experimental huts the mixture provided high levels of mortality against wild pyrethroid resistant *An. arabiensis*; however, the level of increase over CFP or alpha alone was not significant statistically. The degree of improved mortality compared with the individual CFP or alpha components is likely to be influenced by resistance status and mosquito contact time with the insecticides. In Lower Moshi the *An. arabiensis* population was more than 50% susceptible in resistance testing and the alpha ITN in hut remained effective, with 50% mortality. The level of mortality for the mixture (58%) was higher than for CFP or alpha, but slightly lower than the prediction of an additive model. CFP is non-irritant at the dosage used [36] which may favour longer contact times than is usual for a more irritant pyrethroid ITN [38]. The lack of CFP irritancy may be beneficial in terms of mortality as *An. gambiae* requires a contact time of >3 mins to pick up a lethal dosage of CFP. Irritancy caused by alpha in the mixture with CFP may have reduced the mean contact time of *An. arabiensis* with the net, thereby reducing toxicity from CFP in the mixture. In areas of high frequency pyrethroid resistance, the degree of irritancy from alpha is likely to be less, contact time with netting longer, and hence mortality generated by the CFP component would be comparatively greater.

Previous studies in Benin and Tanzania indicated that chlorfenapyr at 100 mg/m^2^ provided little personal protection with 5% blood-feeding inhibition of *An. gambiae s.s.* and 37% for *An. arabiensis* [21], [22]. Conversely in this study CFP produced good rates of *An. arabiensis* feeding inhibition (75%). It was predicted that greater levels of protection could be achieved by mixing CFP with an excito-repellent pyrethroid. The irritant properties of pyrethroids can provide protection even in areas of pyrethroid resistance; the level of protection depends on the species and the level of resistance. In Côte d’Ivoire, for example, alpha 20 mg/m^2^ reduced *An. gambiae* blood-feeding by 84% in an area of high kdr frequency (94% kdr) [39]. In Benin, however, where *An. gambiae* had high frequency L1014F kdr (86%) and elevated expression of cytochrome P450s [40], lambdacyhalothrin (18 mg/m^2^) provided no personal protection, with 82% of *An. gambiae* collected blood-fed [15]. Against highly resistant *Cx. quinquefasciatus* (also in Benin) pyrethroid treated nets continued to provide protection but the level of protection depended on the number of holes per net [41]. In the present study in Tanzania alpha was highly effective in reducing blood-feeding of pyrethroid resistant *Cx. quinquefasciatus* in tunnel tests and *An. arabiensis* in experimental huts. In all cases for resistant and susceptible mosquitoes CFP 100+ alpha 25 produced higher levels of blood-feeding inhibition compared to CFP alone. The relative contribution of alpha or CFP to blood-feeding inhibition will vary according species behaviour and to resistance mechanisms present.

Theoretical models have demonstrated the potential benefits of using insecticide mixtures, based on resistance to each compound being independent and initially rare, with cases of double resistance being extremely rare [42], [43]. In tunnel tests the CFP+alpha mixture produced high levels of mortality against both pyrethroid susceptible and resistant *Cx. quinquefasciatus.* This suggests that mixtures of CFP+alpha are unlikely to place significant selection pressure for pyrethroid resistance on partially pyrethroid resistant populations. Empirical evidence in populations with resistance genes at low to moderate frequency either in experimental huts or in large scale trials is required to determine the effect of mixtures in terms of resistance management.

This study has highlighted the need to adapt testing protocols for the evaluation of new insecticides, particularly determining suitable bioassay exposure times. WHOPES guidelines for evaluation of ITN and LLIN have been developed for testing pyrethroid nets [27], [28]. New insecticides are unlikely to have the same properties of pyrethroids with rapid knock-down and mortality after short exposure times. For CFP a standard 3 mins ball bioassay produced <5%, while in experimental huts mortality was 48%. Clearly 3 mins exposure for CFP nets did not give an indication of field performance. However 30 mins exposure produced 58% mortality, which was closer to actual field performance and may be realistic of actual contact time for a non-irritant insecticide such as CFP on nets in household use. More work is needed comparing bioassay results over a range of exposure times with field performance in experimental huts.

Most malaria vector control programs have failed to implement resistance management strategies, partly due to the dearth of cost-effective ITN and IRS insecticides. Most African countries have relied upon repeated IRS spraying with pyrethroids or DDT concurrent with the mass distribution of pyrethroid LLINs [1]. Such practice is likely to accelerate resistance and WHO has since recommended that pyrethroid IRS should not be used in areas of high pyrethroid LLIN coverage. For optimal use of insecticide mixtures for delaying the selection of resistance: (1) the insect should not be resistant to both components; (2) the combination must maintain its integrity over time, with the components showing similar decay rates; and (3) the modes of resistance must be unique [44]. CFP and alpha have unique modes of action; LLIN versions of CFP and alpha mixtures should be developed to maintain integrity of both components for long-lasting malaria control and resistance management.

As a combination net, a mixture of CFP and alpha provides a number of advantages over a pyrethroid only net. A combination of CFP and alpha should be effective in reducing the longevity of pyrethroid resistant and susceptible *An. gambiae* malaria vectors regardless of the frequency of pyrethroid resistance in the population. It would provide personal protection for users. It may have benefits of resistance management, particularly in areas of pyrethroid susceptibility or areas with a low frequency of pyrethroid resistance. It should be effective in places where more than one vector species coexist or where one species is resistant to pyrethroid and one is not.
