# Correction: ITN Mixtures of Chlorfenapyr (Pyrrole) and Alphacypermethrin (Pyrethroid) for Control of Pyrethroid Resistant *Anopheles arabiensis* and *Culex quinquefasciatus*

**DOI:** 10.1371/annotation/9c1e150f-b310-45b9-b835-220e15489781

**Published:** 2014-01-16

**Authors:** Richard M. Oxborough, Jovin Kitau, Johnson Matowo, Emmanuel Feston, Rajab Mndeme, Franklin W. Mosha, Mark W. Rowland

The titles for Table 2 and 3 were switched. 

The title for Table 2 should read, "Table 2. Resistance status of Cx. quinquefasciatus TPRI strain. Percentage mortality of Cx. quinquefasciatus TPRI strain after exposure in cylinder bioassays lined with treated papers at diagnostic concentrations."

The title for Table 3 should read, "Table 3. Summary of Bottle Bioassay Results; KDT-50 and Resistance Ratios for Cx. quinquefasciatus TRI (susceptible) and Muheza strains (resistant). Time taken (minutes) for 50% of mosquitoes to be knocked-down (KDT-50) during exposure in 250 ml glass bottles treated with 10 mg/ml permethrin alone (no synergist) or mixed with synergists PBO, DEF or PBO+DEF (n = 100 for each treatment)."

